# Melhorando as Metanálises

**DOI:** 10.36660/abc.20230331

**Published:** 2023-06-19

**Authors:** Fernando Mendes Sant’Anna, Mariana Bonacossa Sant’Anna, Lucas Bonacossa Sant’Anna

**Affiliations:** 1 Universidade Federal do Rio de Janeiro Macaé RJ Brasil Universidade Federal do Rio de Janeiro, Campus Macaé, Macaé, RJ – Brasil; 2 Hospital Santa Izabel Cabo Frio RJ Brasil Hospital Santa Izabel, Cabo Frio, RJ – Brasil; 3 Fundação Técnico-Educacional Souza Marques Rio de Janeiro RJ Brasil Fundação Técnico-Educacional Souza Marques (FTESM), Rio de Janeiro, RJ – Brasil

**Keywords:** Infarto do Miocárdio, Intervenção Coronária Percutânea, Metanálise, Estatística, Análise de Dados, Idoso, Tratamento Conservador/tendências

A metanálise (MA) de Meng-jin et al.,^1^ publicada nesta edição da revista, traz informações importantes sobre o tratamento invasivo de pacientes idosos (≥ 75 anos) com infarto agudo do miocárdio sem supradesnivelamento do segmento ST (IAMSSST) versus tratamento conservador.

Mesmo ciente dos benefícios da revascularização precoce em pacientes idosos e jovens,^2^ no primeiro grupo, sabe-se que a preocupação com os riscos de complicações em procedimentos invasivos reduz o número de intervenções nesse grupo.^3^ Por outro lado, com o rápido crescimento da população idosa no mundo, a Organização Mundial da Saúde prevê um aumento significativo da mortalidade por doença arterial coronariana nas próximas décadas,^4^ tornando-se essencial desenvolver estratégias de tratamento eficazes em pacientes idosos com IAMSSST.

Os autores realizaram uma extensa pesquisa em várias bases de dados e terminaram por incluir 27 estudos em sua análise, 5 dos quais foram randomizados e 22 observacionais. Os desfechos primários foram morte por todas as causas, infarto do miocárdio (IM), acidente vascular cerebral e sangramento maior. Os desfechos secundários incluíram efeitos cardiovasculares adversos maiores (MACE), morte cardíaca, revascularização e readmissão.

No entanto, a metodologia utilizada pelos autores foi o mais nos chamou a atenção neste interessante artigo. Além das ferramentas clássicas utilizadas na MA, os autores também empregaram um recurso denominado *trial sequence analysis* (TSA), que, embora útil, é muito pouco conhecido pela maioria dos pesquisadores. A análise sequencial é um método estatístico no qual o número final de pacientes analisados não é predeterminado, mas a amostragem ou inscrição de pacientes é decidida por uma regra de parada predeterminada, como a satisfação de uma significância estatística. Assim, os investigadores podem concluir antes dos métodos estatísticos tradicionais, reduzindo tempo, custo, esforço e recursos.^5^

MAs adequadamente conduzidas são consideradas as melhores evidências na literatura científica. No entanto, as MAs estão expostas a resultados significativamente enganosos (erros tipo I; α) ou resultados erroneamente insignificantes (erros tipo II; β) causados por ensaios de baixa qualidade ou com potência inadequada, viés de publicação e testes de significância repetidos.^6^

**Figura 1:**
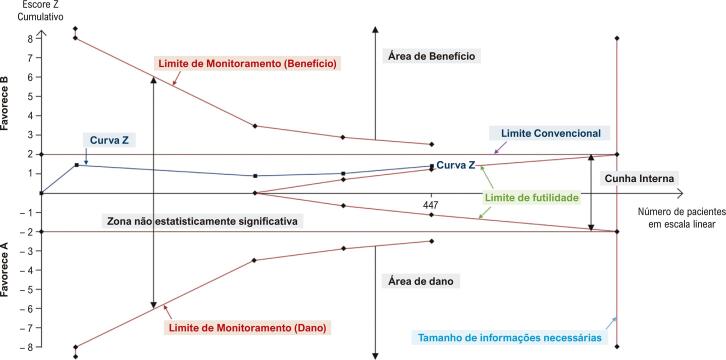
– Gráfico de análise sequencial experimental. O gráfico apresenta limites de monitoramento, limites de futilidade, limites convencionais e tamanho de informações necessárias. O gráfico é dividido pelo limite de monitoramento e limite de futilidade em quatro zonas: área de benefício, área de dano, cunha interna e zona não estatisticamente significativa. Adaptado de Kang H.5

TSA é um método de MA cumulativo desenvolvido para pesar os erros α e β enquanto estima quando o efeito é grande o suficiente para ser improvável de ser afetado por estudos posteriores.^6^ O TSA é exibido como um gráfico cartesiano com um escore z cumulativo no eixo y e o número de pacientes no eixo x, subdividido em quatro zonas por quatro linhas: limites de monitoramento para benefícios e danos e dois limites de futilidade (Figura 1). Duas linhas paralelas ao eixo x geralmente são exibidas, mostrando a linha estatisticamente significativa convencional em z, correspondendo a 1,96. TSA é geralmente usada em ensaios clínicos randomizados (ECR).

A linha estatística cumulativa z é construída sequencialmente, adicionando um estudo com critérios cronológicos.^7^ O final da linha corresponde ao último estudo adicionado. Ele ficará em uma das seguintes zonas: “benefício”, “dano”, “cunha interna” ou “não significativo estatisticamente”, representando um resultado estatisticamente significativo para as duas primeiras áreas (“benefício” e “dano”) ou fortes evidências de que estudos posteriores dificilmente conseguirão alterar os resultados sem efeito (área de “cunha interna”). A presença na área “não estatisticamente significativa” significa que mais estudos são necessários.

No estudo de Meng-jin et al.,^1^ a TSA revelou que informações suficientes dos ECRs foram obtidas apenas para os desfechos de IM, MACE e revascularização, mas não para outros desfechos, provavelmente devido a um número insuficiente de pacientes.^1^ Portanto, os autores decidiram adicionar estudos observacionais à revisão para aumentar o tamanho da amostra e diminuir o viés o máximo possível. Isso permitiu mostrar um efeito positivo do tratamento invasivo em quase todos os parâmetros e apenas um efeito negativo: o aumento do sangramento no subgrupo de pacientes ≥ 85 anos.

Este estudo tem algumas limitações, e os autores as discutem brevemente, como diferentes formas de tratamentos invasivos (ICP ou CABG) e diferentes definições de resultados. Outra limitação, não mencionada diretamente, diz respeito à combinação de estudos randomizados com observacionais.^8^ Mesmo após o ajuste multivariado, sabemos que são dois tipos de estudos clínicos muito diferentes, e sempre é preciso cautela na interpretação dos resultados. Estudos randomizados continuam sendo o padrão-ouro e devem sempre guiar nossa prática, embora às vezes seja desejável incorporar estudos observacionais em uma MA.

Assim, podemos concluir que a MA de Meng-jin et al.^1^ confirma os achados de alguns ECR e estudos observacionais, mas talvez ainda mais importante do que isso, nos lembra de recursos muito interessantes que podemos (e devemos) usar quando decidimos realizar uma MA.
