# Supplementary material for: Melhorando as Metanálises
Source: Arq Bras Cardiol. 2023 Jun 19;120(6):e20230331. [Article in Portuguese] doi: 10.36660/abc.20230331 (PMC10344416; doi:10.36660/abc.20230331)
Supplement: Translation

# Translation: Improving Meta-analyses

**Authors:** Fernando Mendes Sant’Anna, Mariana Bonacossa Sant’Anna, Lucas Bonacossa Sant’Anna

**Affiliations:** 1 Brazil Universidade Federal do Rio de Janeiro, Campus Macaé, Macaé, RJ – Brazil; 2 Brazil Hospital Santa Izabel, Cabo Frio, RJ – Brazil; 3 Brazil Fundação Técnico-Educacional Souza Marques (FTESM), Rio de Janeiro, RJ – Brazil

**Keywords:** Myocardial Infarction, Percutaneous Coronary Intervention, Meta-Analysis, Statistics, Data Analysis, Aged, Conservative Treatment/trends

The meta-analysis (MA) by Meng-jin et al.,^1^ published in this issue of the journal, brings important information about the invasive treatment of elderly patients (≥ 75 years old) with acute myocardial infarction without ST-segment elevation (NSTEMI) versus conservative treatment.

Even aware of the benefits of early revascularization in both elderly and young patients,^2^ in the first group, the concern with the risks of complications in invasive procedures are known to reduce the number of interventions in this group.^3^ On the other hand, with the rapid growth of the elderly population in the world, World Health Organization predicts a significant increase in mortality from coronary artery disease in the coming decades,^4^ making it essential to develop effective treatment strategies in elderly patients with NSTEMI.

The authors conducted extensive research in several databases and finished by including 27 studies in their analysis, 5 of which were randomized and 22 observational. The primary outcomes were all-cause death, myocardial infarction (MI), stroke, and major bleeding. Secondary outcomes included major adverse cardiovascular effects (MACE), cardiac death, revascularization, and readmission.

However, the methodology used by the authors was what most caught our attention in this interesting article. In addition to the classic tools used in the MA, the authors also employed a resource called *trial sequential analysis* (TSA), which, although useful, is very little known by most researchers. Sequential analysis is a statistical method in which the final number of patients analyzed is not predetermined, but sampling or enrollment of patients is decided by a predetermined stopping rule, such as satisfying a statistical significance. Accordingly, the investigators may conclude earlier than traditional statistical methods, reducing time, cost, effort, and resources.^5^

Adequately conducted MAs are considered the best evidence in the scientific literature. Nonetheless, MAs are exposed to significantly misleading results (type I errors; α) or erroneously insignificant results (type II errors; β) caused by low-quality or inadequately powered trials, publication bias, and repeated significance testing.^6^

TSA is a cumulative MA method developed to weigh α and β errors while estimating when the effect is large enough to be unlikely to be affected by further studies.^6^ TSA is displayed as a Cartesian graph with a cumulative z-score on the y-axis and the number of patients on the x-axis, subdivided into four zones by four lines: monitoring boundaries for benefit and harm and two futility boundaries (Figure 1). Two lines parallel to the x-axis are usually displayed, showing the conventional statistically significant line at z, corresponding to 1.96. TSA is generally used in randomized clinical trials (RCT).

**Figure 1:** – Trial sequential analysis graph. The graph presents monitoring boundaries, futility boundaries, conventional boundaries, and required information size. The graph is divided by monitoring boundary and futility boundary into four zones: area of benefit, area of harm, inner wedge, and not statistically significant zone. Adapted from Kang H.5

The cumulative z statistic line is constructed sequentially, adding a study with chronological criteria.^7^ The end of the line corresponds to the lastly added study. It will lie in one of the following zones: “benefit,” “harm,” “inner wedge,” or “not statistically significant,” representing a statistically significant result for the first two areas (“benefit” and “harm”) or strong evidence that further studies will hardly be able to change the no-effect results (“inner wedge” area). Presence in the “not statistically significant” area means that further studies are needed.

In the study by Meng-jin et al.,^1^ the TSA revealed that sufficient information from the RCTs was obtained only for the MI, MACE, and revascularization outcomes but not for other outcomes, probably due to an insufficient number of patients.^1^ Therefore, the authors decided to add observational studies to the review to increase the sample size and decrease bias as much as possible. This allowed to show a positive effect of the invasive treatment in almost all parameters and only one negative effect: the increase in bleeding in the subgroup of patients ≥ 85 years.

This study has some limitations, and the authors discuss them briefly, such as different forms of invasive treatments (PCI or CABG) and different definitions of outcomes. Another limitation, not mentioned directly, concerns combining randomized with observational studies.^8^ Even after multivariate adjustment, we know these are two very different types of clinical studies, and caution is always needed when interpreting the results. Randomized studies remain the gold standard and should always guide our practice, although sometimes it is desirable to incorporate observational studies in a MA.

Thus, we can conclude that Meng-jin ey al.^1^ MA confirms the findings of some RCT and observational studies, but perhaps even more important than that, it reminds us of very interesting resources that we can (and should) use when we decide to conduct a MA.
